# Supplementary material for: Age-Adjusted Cerebellar Volumetry and Developmental Outcomes in Extremely Low Birth Weight Infants
Source: bioRxiv. 2026 Oct 1:2026.09.29.755417. Preprint. [Version 1] doi: 10.64898/2026.09.29.755417 (PMC13648623; doi:10.64898/2026.09.29.755417)
Supplement: Supplementary file 1 [file media-1.pdf]

Supplementary material

Table S1 Robustness of the three-level PMA-by-outcome interaction

**Analysis**	**Full cohort (n = 42)**	**PMA 36–46 weeks (n = 29)**
Conventional joint interaction P	0.002404	0.010739
HC3 robust joint interaction P	0.142532	0.078929
Leave-one-out interaction P range	0.000281–0.176358	0.003197–0.147010

HC3 tests use heteroskedasticity-robust covariance estimates. Leave-one-out analyses refit the model after omitting each observation in turn; corresponding sample sizes are 41 and 28. All 42 confirmed developmental classifications were retained in the primary cohort.

Table S2 Group-specific cross-sectional PMA–volume slopes in the 38–42-week subset

**Developmental outcome**	**n**	**Slope (95% CI), cm^3/week**
Normal development	4	2.55 (-0.31 to 5.42)
Isolated or specific impairment	5	5.09 (1.66 to 8.52)
Moderate/severe impairment	5	1.75 (-0.90 to 4.40)

PMA boundaries are inclusive. All 14 infants were also included in the 36–46-week subset and full cohort. Slopes represent between-infant associations, not within-infant growth. Confidence intervals use conventional model standard errors. Omnibus interaction: $F(2, 8) = 1.62$, $P = 0.256$; model $R^2 = 0.778$.
